# Bladder carcinosarcoma treated by cystectomy and adjuvant chemotherapy with good outcomes: a case report

**DOI:** 10.1186/s13256-023-04028-3

**Published:** 2023-07-19

**Authors:** Fadila Kouhen, Zineb Dahbi, Mohammed Afif, Meriem Chihabeddine, Nadia Errafiy, Kenza Oqbani, Youssef Omor, Amal Elhossini

**Affiliations:** 1 Department of Radiotherapy, International University Hospital Sheikh Khalifa, Mohammed VI University of Health Sciences (UM6SS), Casablanca, Morocco; 2 Department of Radiotherapy, Faculty of Medicine of Tangier, Abdelmalek Essaadi University, Tangier, Morocco; 3 National Reference Laboratory (LNR), Mohammed VI University of Health Sciences (UM6SS), Casablanca, Morocco; 4 Pathology Laboratory, International University Hospital Sheikh Khalifa, Mohammed VI University of Health Sciences (UM6SS), Casablanca, Morocco; 5 Department of Radiology, National Institute of Oncology, Rabat, Morocco; 6 Pathology Laboratory PATHONORD, Tangier, Morocco

**Keywords:** Bladder carcinosarcoma, Cystectomy, Chemotherapy, Outcomes

## Abstract

**Background:**

Primary carcinosarcoma of the bladder is a rare and highly aggressive tumor, representing less than 1% of all bladder neoplasms. There is no specific treatment guideline has for carcinosarcoma of the bladder, and majority of published patients was treated exclusively by surgery.

**Case presentation:**

We report a case of 65-year-old Moroccan man, presented with macroscopic hematuria, pollakiuria and painful urination. Histological analysis showed a biphasic epithelial and mesenchymal proliferation, with invasion of lamina propria and muscularis, compatible with diagnosis of bladder carcinosarcoma. The patient was treated with cystectomy and adjuvant chemotherapy based on gemcitabin-cisplatin, 18 months after treatment, patient still free of recurrence.

**Conclusion:**

Carcinosarcoma of the urinary bladder is a rare and aggressive tumor regardless treatment. A multidisciplinary management based on radical cystectomy and combined adjuvant treatments can improve prognosis. In this work, we suggest to propose adjuvant chemotherapy whenever possible.

## Background

Primary bladder carcinosarcoma (CS) is an aggressive and extremely rare form of bladder cancer representing about 0.1–0.3% of all urinary bladder malignancies [1].

Carcinosarcomas are defined by the World Health Organization as a biphasic tumor consisting of malignant epithelial and mesenchymal components. They predominantly affect elderly patients with an average age of 70 years [2]. Due to the rarity of this tumor, an optimal treatment has not yet been defined, and prospective studies still do not exist to allow therapeutic standardization. We report a case of a locally advanced CS of the bladder successfully treated with cystectomy and adjuvant chemotherapy. This case has been reported in line with the CARE guidelines [3].

## Case presentation

A 65-year-old Moroccan man with a history of diabetes and cigarette smoking had no relevant exposure to chemical industrial products in the past, and no notable surgical or family history.

The patient presented 3 months before his medical consult with macroscopic hematuria, pollakiuria, and painful urination, without acute urine retention or digestive disorders, evolving in a context of conservation of the general state. Physical examination did not reveal any abnormalities. No mass was palpable on clinical examination of the abdomen.

**Fig. 1:**
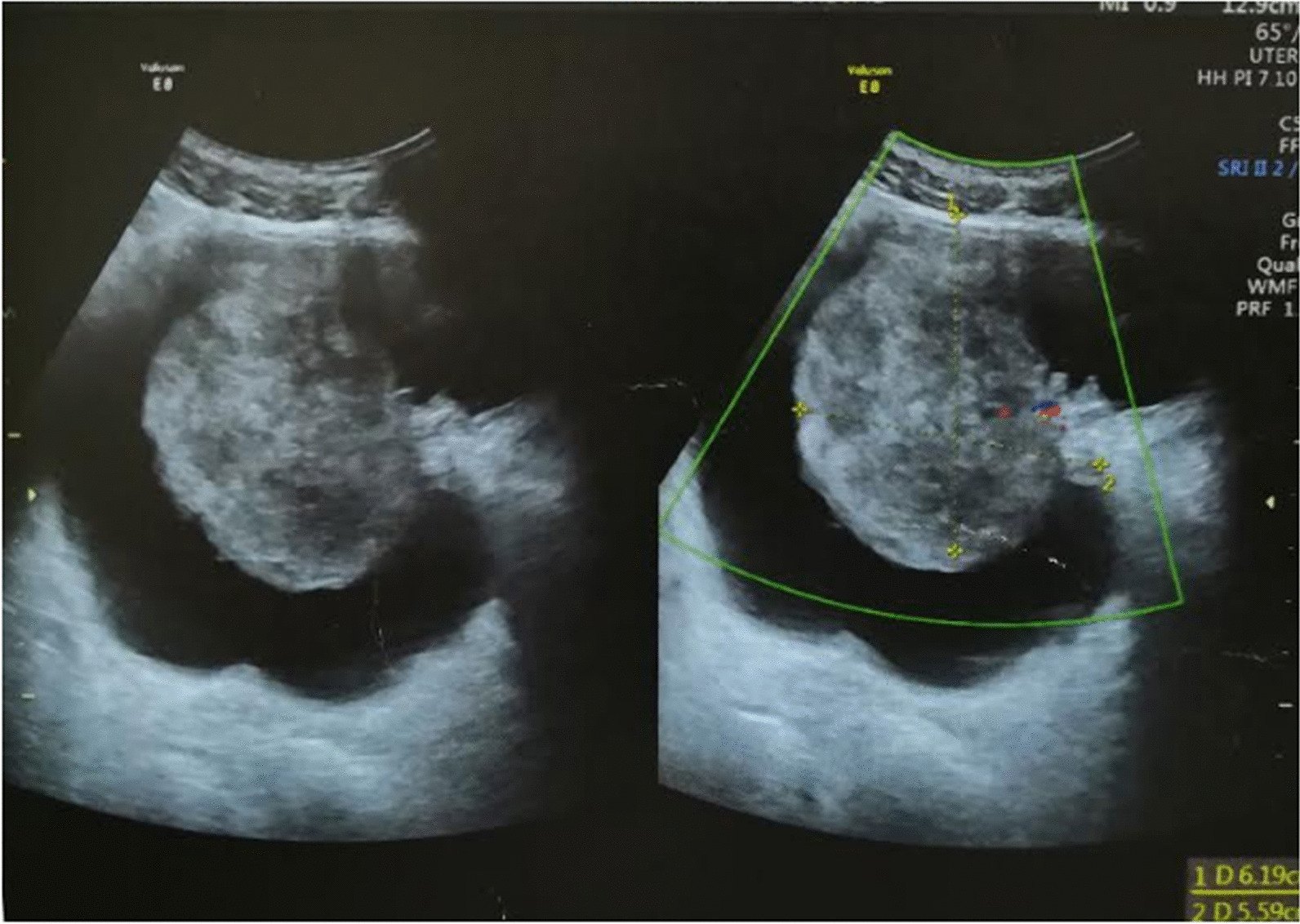
Pelvic ultrasound showed an echogenic mass of the left lateral wall of bladder

Pelvic ultrasound showed an echogenic irregular mass on the left lateral wall of the bladder associated with soft dilatation of the upper urinary tract (Fig. 1). The baseline blood workup, including NSF and Glomerular Filtration Rate, did not reveal any abnormality.

Cystoscopy revealed a non-papillary massive mass on the left lateral wall of the bladder, and a transurethral resection of the bladder tumor (TURBT) was performed at the same time. Pelvic MRI was not done due to lack of means.

Histopathological analysis of the resected tissues showed a biphasic epithelial and mesenchymal proliferation, with invasion of the lamina propria and muscularis, compatible with the diagnosis of bladder carcinosarcoma, stage pT2. The transurethral resection was complete.

**Fig. 2:**
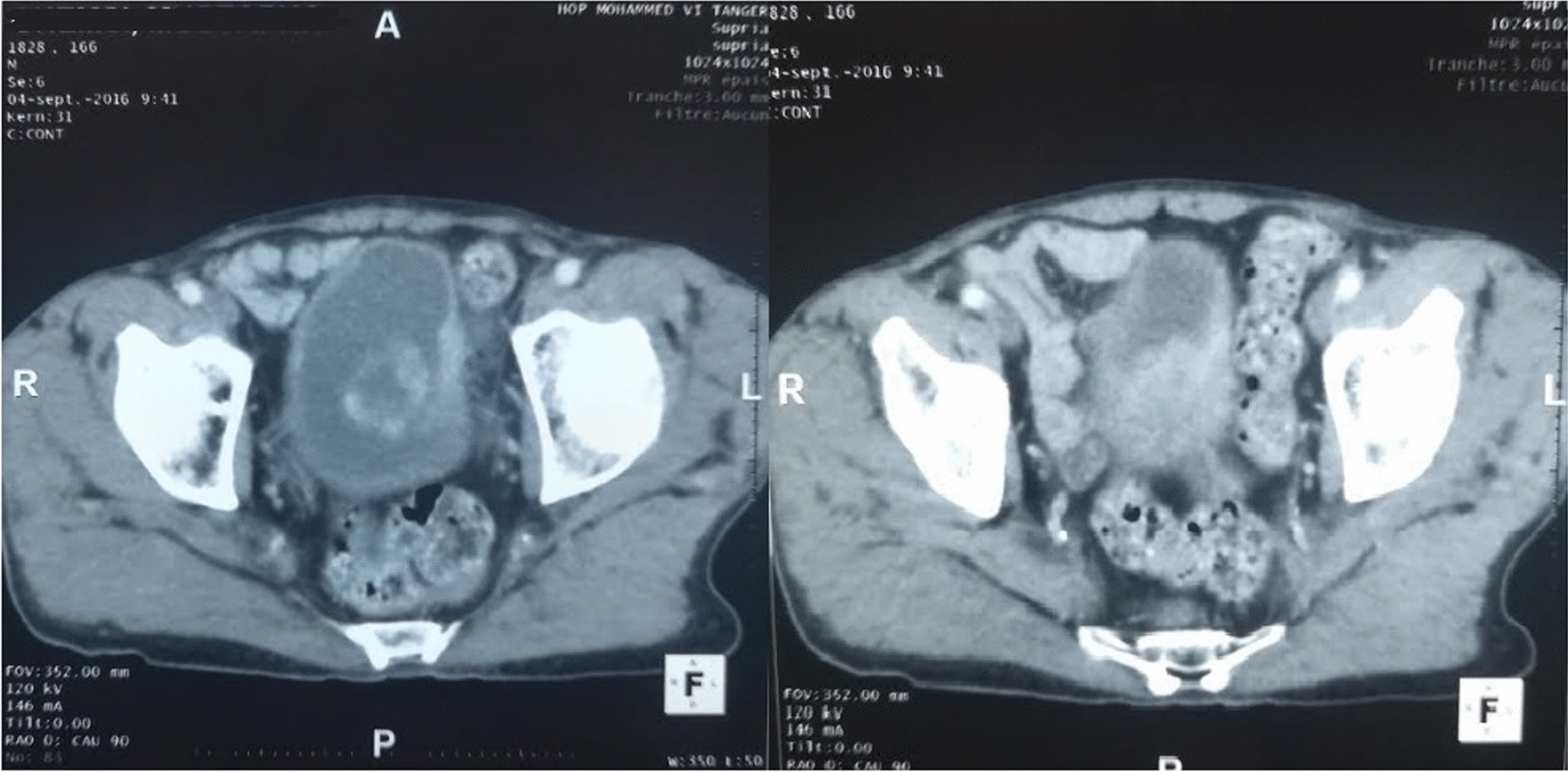
Pelvic computed tomography scan after injection of contrast showing an heterogeneous mass, invading the bladder wall until serosa

Staging including pelvic CT scan revealed a heterogeneous mass of the dome and the left wall of the bladder, measuring 10 × 6 cm, invading the bladder wall until serosa (Fig. 2). There were no iliac lymph nodes, the ipsilateral ureteral orifice was intact, and there weren't any signs of upper urinary tract obstruction. Thoraco-abdominal CT, as well as bone scan, were free of metastasis. The patient was classified as T3N0M0.

**Fig. 3:**
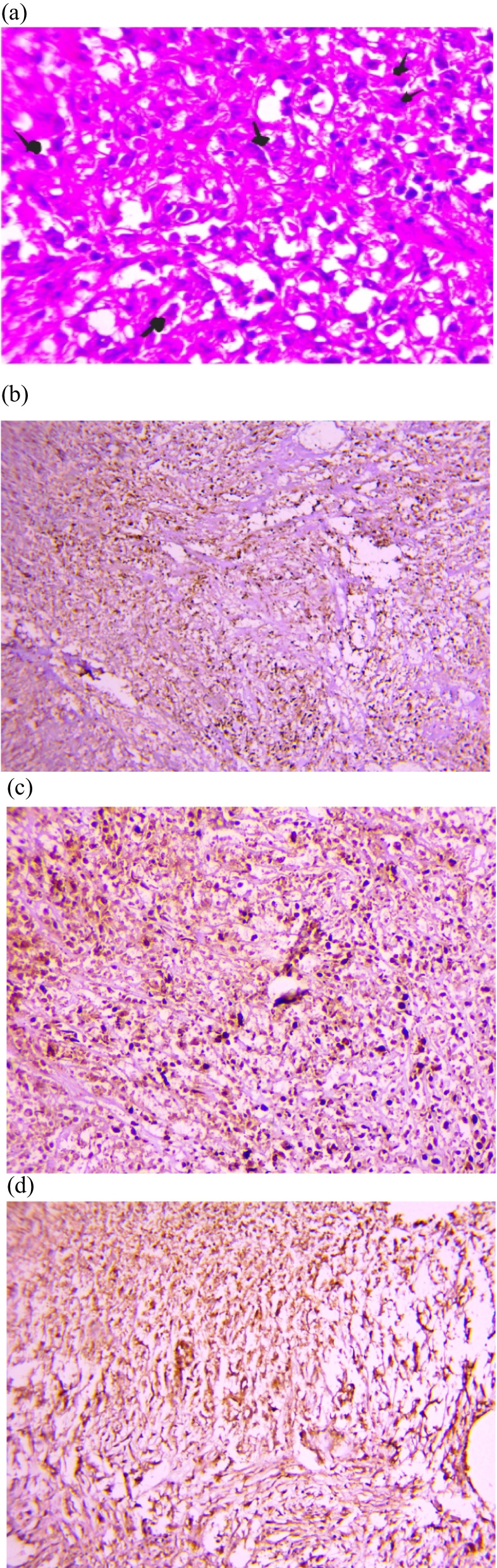
Histopathology of the tumor: **a** Microscopic view of the tumor with epithelial and sarcomatoid components (H-E × 40). **b** Immunohistochemical staining showing positivity of Pancytokeratin (CK AE1/AE3) (H-E × 10). **c** Immunohistochemical staining showing positivity of Vimentin (HE, Gx20). **d** Immunohistochemical staining showing positivity of GATA3 (H-E × 20)

After urology tumor board meeting, the patient underwent radical cystoprostatectomy with ileal conduit urinary diversion (Bricker), and an extended pelvic lymph node dissection. There were no post-operative complications. Histopathological examination of the specimen revealed a high-grade carcinosarcoma invading the bladder wall until the serosa and the trigone, with the prostate and the urethra free from tumor extension. Two of eight pelvic lymph nodes were positive for metastatic carcinosarcoma without extra-nodal extension. All surgical margins were negative for tumor. Immunohistochemistry showed positivity of Pan-cytokeratin, GATA3, and vimentin, and negativity for CK7, myogenin, desmin, and PSA (Fig. 3).

After discussion with the multidisciplinary board, an adjuvant chemotherapy based on gemcitabine-cisplatin was indicated, and a total of six cycles were received with no significant toxicity. The patient was scheduled for trimestrial clinical visits, Cystoscopy · months after finishing treatment, UroTDM performed each semester. Twelve months after treatment, the patient is still under follow-up with no locoregional or metastatic disease.

## Discussion

Carcinosarcomas (CS) of the bladder and urinary tract are rare and extremely aggressive tumors [2]. The incidence of CS is 0.1 to 0.3 of all bladder malignancies. In the SEER database, Wright *et al*. identified only 135 cases of SC and 166 cases of CS of the bladder from a total of 182,283 patients identified with primary bladder cancer. These tumors are more common in males (male–female ratio 1.9/1), and the incidence increases with age [1].

Considering tumor's aggressiveness, approximately 85% of patients with CS present with MIBC and metastatic disease at diagnosis [2].

Carcinosarcomas are biphasic tumors simultaneously containing malignant epithelial and malignant mesenchymal tissue components. Their histogenesis remains uncertain; however, some theories suggest that these tumors might develop as a result of undifferentiated, totipotent neoplastic cells that undergo multiple pathways of differentiation into either mesenchymal or epithelial elements [3].

There are no specific risk factors for the development of carcinosarcoma of the bladder. Nonetheless, bladder schistosomiasis is a risk factor for bladder cancer of different histological types, including sarcomatoid carcinoma variants. Srougi and al reported a very rare case of a young patient from central Africa diagnosed with carcinosarcoma of the bladder following local schistosomiasis infection [4]. The authors stated that the inflammation/carcinogenic sequence induced by the parasite could have possibly generated such an aggressive rare neoplastic variant.

Presenting symptoms in CS are similar to those of other types of bladder cancer, the most common is painless hematuria; other signs can be observed in some patients including dysuria and urinary retention. Our patient presented an isolated macroscopic hematuria without other urinary signs. The initial steps in the diagnostic and treatment strategy do not differ from the workup of other bladder tumors. Furthermore, cystoscopy remains the gold standard for diagnostic evaluation of hematuria and assessment of intravesical papillary masses [5].

Currently, optimal management of bladder CS has not been clearly defined because of its rarity, there are no adequate randomized prospective studies for therapeutic standardization [6]. Consequently, the treatment of bladder SC has usually been extrapolated from the approach for patients with urothelial carcinoma; however, several cases reports and a few retrospective studies have provided some insight into therapy for bladder carcinosarcoma [7].

The main treatment of bladder carcinosarcoma consists of radical cystectomy associated with pelvic lymphadenectomy. Neoadjuvant chemotherapy has been used in many cases, with some complete responses. Neoadjuvant regimens usually used were GC (cisplatin/gemcitabine), AI (Adriamycin/ifosfamide), and MVAC (methotrexate/vinblastine/Adriamycin/cisplatin). However, we can't conclude the benefit of neoadjuvant treatment because of the rarity of cases [8].

Adjuvant chemotherapy may be used after radical surgery if the patient did not receive neoadjuvant treatment [9]. The potential benefit of adjuvant chemotherapy is to eradicate microscopic residual disease and increase the chances for long-term cancer control. For our patient, we chose an adjuvant protocol based on 6 cycles of gemcitabine/cisplatin.

Moreover, Zachariadis *et al*. reported a very interesting case report of a 76-year-old female with a past medical history of heart failure and non-insulin-dependent diabetes treated with transurethral resection followed by radiotherapy with adverse final outcome. The authors concluded that radiotherapy alone is insufficient as a treatment option for these aggressive tumors [10].

Carcinosarcomas of the urinary bladder have a poor prognosis regardless of treatment modalities, and the majority of patients die within the first year. In the large data base of the Surveillance, Epidemiology, and End Results (SEER), the pathological stage was identified as the principal predictor factor of survival. Patients with regional and distant spread of disease had a twofold and eightfold increased risk of mortality [10].

However, Akoluk *et al*. reported the case of a 79-year-old patient treated by radical cystectomy and who did not receive any form of adjuvant treatment post-operatively with a long-term disease-free 2-year follow-up period [11].

## Conclusions

Carcinosarcoma of the urinary bladder is a rare and highly aggressive tumor that poses significant challenges in its management. Despite various treatment modalities, the prognosis of bladder carcinosarcoma remains poor. However, a multidisciplinary approach that includes radical cystectomy, which involves the surgical removal of the bladder, along with combined adjuvant treatments, has shown potential to improve prognosis.

Based on the findings from this case, we strongly recommend considering adjuvant chemotherapy as part of the treatment plan whenever possible for this aggressive histologic subtype. Adjuvant chemotherapy, which is administered after surgery, aims to eradicate any residual disease at the microscopic level and may increase the chances of long-term cancer control. By incorporating adjuvant chemotherapy into the management approach, we may be able to enhance the effectiveness of treatment for bladder carcinosarcoma and potentially improve patient outcomes.

## Data Availability

Not applicable.

## Abbreviations

CS: Carcinosarcoma
CK: Cytokeratin
GC: Gemcitabin-Cisplatin
AI: Adriamycin-Ifosfamide
